# Community management of acute malnutrition (CMAM) programme in Pakistan effectively treats children with uncomplicated severe wasting

**DOI:** 10.1111/mcn.12623

**Published:** 2018-11-29

**Authors:** Víctor M. Aguayo, Nina Badgaiyan, Syed Saeed Qadir, Ali Nasir Bugti, Muhammad Mazhar Alam, Noureen Nishtar, Melanie Galvin

**Affiliations:** ^1^ Programme Division United Nations Children's Fund (UNICEF) New York NY USA; ^2^ UNICEF, Regional Office for South Asia Kathmandu Nepal; ^3^ UNICEF, Country Office for Pakistan Islamabad Pakistan; ^4^ Nutrition Program, Department of Health Government of Balochistan Quetta Pakistan; ^5^ Concern Worldwide, Country Office for Pakistan Islamabad Pakistan; ^6^ Action Contre la Faim (ACF) International, Country Office for Pakistan Islamabad Pakistan

**Keywords:** community management of acute malnutrition (CMAM), mid‐upper arm circumference (MUAC), Pakistan, severe acute malnutrition (SAM), severe wasting, South Asia

## Abstract

Severe wasting is the most widespread form of severe acute malnutrition, affecting an estimated 17 million children globally. This analysis assesses the effectiveness of Pakistan's community management of acute malnutrition (CMAM) programme. We conducted a retrospective case series analysis of 32,458 children aged 6–59 months who were admitted to the programme with a mid‐upper arm circumference (MUAC) < 115 mm (January 1–December 31, 2014). We found that at admission, 59.6% of the children were girls and 87.4% were in the age group 6–23 months old. While in the programme, 120 children (0.4%) died, 3,456 (10.6%) defaulted, and 28,882 (89.0%) were discharged after a mean length of stay of 69.3 ± 25.7 days. Children's mean weight gain while in the programme was 3.2 ± 2.7 g/kg body weight/day. At discharge, 28,499 children (98.7% of discharged) had recovered (MUAC ≥ 125 mm). The odds of death were significantly higher among children with weight‐for‐height (WHZ) < −3 and/or height‐for‐age (HAZ) < −2 at admission. The odds of recovery on the basis of MUAC ≥125 mm were higher among children with HAZ ≥ −2 at admission. The odds of recovery on the basis of WHZ ≥ −2 were significantly higher among children with WHZ ≥ −3 and/or HAZ < −2 at admission. Pakistan's CMAM programme is effective in achieving good survival and recovery rates. Population‐level impact could be increased by giving priority to children 6–23 months old and children with multiple anthropometric failure and by scaling up CMAM in the provinces and areas where the risk, prevalence, and/or burden of severe acute malnutrition is highest.

**Key messages**

- In 2014, 32,458 children aged 6–59 months were admitted to Pakistan's CMAM programme (MUAC < 115 mm): 60% of the children were girls, and 87% were in the age group 6–23 months old.
- While in the programme, 120 children (0.4%) died, 3,456 (10.6%) defaulted, and 28,882 (89.0%) were discharged. At discharge, 98.7% of the children had recovered.
- Pakistan's CMAM programme is effective in achieving good survival and recovery rates. Population‐level impact could be increased by scaling up CMAM in the provinces/areas where the risk, prevalence, and/or burden of severe acute malnutrition is highest.

**Abbreviations**
CMAM: Community management of acute malnutrition
HAZ: Height‐for‐age *z*‐score
MUAC: Mid‐upper arm circumference
NGO: Non‐governmental organization
OTP: Outpatient therapeutic programme
SAM: Severe acute malnutrition
SC: Stabilization centre
WHZ: Weight‐for‐height *z*‐score

## 1 INTRODUCTION

Severe wasting is the most widespread form of severe acute malnutrition (SAM), affecting an estimated 16.4 million children globally (UNICEF, World Health Organization (WHO), & World Bank Group, 2018). Severe wasting in children aged 6–59 months is defined as a weight‐for‐height z‐score (WHZ) < −3 or a mid‐upper arm circumference (MUAC) < 115 mm (WHO & UNICEF, 2009). Severe wasting is driven by poor maternal nutrition, poor complementary feeding practices, seasonal food insecurity, environmental enteropathy, and chronic/acute infections (Trehan & Manary, 2015).

Mortality rates in severely wasted children are 9 times higher than in children who are not wasted (Black et al., 2008). Therefore, the early detection and treatment of severe wasting is an evidence‐based nutrition intervention that improves child survival, growth, and development (WHO, 2013a). The WHO and UNICEF recommend that children with medically complicated severe wasting be treated as inpatients in a facility, whereas children with medically uncomplicated severe wasting should be treated at home with the support of a community‐based programme for the management of acute malnutrition (CMAM; WHO, 2013b). When active case finding and early detection strategies are in place, 90–95% of affected children can be treated in the community using the CMAM approach (Collins et al., 2006). Further, programmatic evidence supports the use of ready‐to‐use therapeutic foods (RUTF) in CMAM programmes (Collins, 2004). The shift from facility‐based care to community‐based care combined with the effectiveness of RUTF has allowed CMAM programmes to grow rapidly, treating an increasing number of children cost‐effectively (Bhutta et al., 2013).

The majority of children suffering from severe wasting live in Asia. In 2014, five Asian countries were home to about 12 million children with severe wasting (i.e., ~70% of the global burden; Ahmed et al., 2014). Pakistan is at the centre of this crisis. The latest surveys indicated that the proportion of children aged 0–59 months who were wasted ranged between 10.8% and 15.1% whereas the proportion of children who were severely wasted ranged between 3.3% and 5.8% (Government of Pakistan, 2013; National Institute of Population Studies, 2013). In 2014, it was estimated that 3.3 million children aged 0–59 months were wasted and 1.2 million were severely wasted (Ahmed et al., 2014; Government of Pakistan, 2014).

The Government of Pakistan has adopted CMAM to provide care for children with acute malnutrition. National guidelines were initially developed in 2010 and were subsequently updated in 2015 as new evidence emerged (Government of Pakistan, 2014). They provide technical guidance for the management of acute malnutrition both in government‐led programmes and in those supported by development partners and non‐governmental organizations (NGOs).

The aim of this analysis is to assess the effectiveness of Pakistan's CMAM programme in providing care for children with severe wasting. The objectives are (a) to estimate death, default, discharge, and recovery rates among children admitted to the programme between January 1 and December 31, 2014; (b) to estimate mean weight gain, length of stay, and number of follow‐up visits among such children as a function of their nutritional status at admission and the discharge criteria used; and (c) to make recommendations—on the basis of our findings—for the design and implementation of programmes for children with SAM in Pakistan and South Asia.

## 2 METHODS

Pakistan's CMAM programme encompasses three components: (a) early detection of children with SAM through community outreach and active case finding to maximize coverage and impact; (b) inpatient therapeutic care in a stabilization centre (SC) for children with medically complicated SAM; and (c) outpatient therapeutic care through the outpatient therapeutic programme (OTP) for children with medically uncomplicated SAM.

### 2.1 Early detection through community outreach

Wherever the CMAM programme is implemented, the detection of children with SAM is systematically ensured by health workers, which include *Lady Health Workers* and *Community Midwives*, sometimes supported by volunteers. Where NGOs operate, community outreach workers are often recruited to assist with case finding and follow‐up. Health workers and community outreach workers systematically screen children at paediatric wards and health facilities, through health campaigns, outreach programmes, child growth monitoring sessions, house‐to‐house visits, and other opportunities.

### 2.2 Inpatient therapeutic care at SCs

Children with (a) bilateral pitting oedema +++, (b) children with MUAC < 115 mm and poor/no appetite, and (c) children with MUAC < 115 mm and medical complications are referred to the SC where they are treated following guidelines for the management of SAM based on those by WHO (2013c). Once stabilized (i.e., oedema resolves, medical complications are treated, appetite returns, and weight gain starts), children are discharged to the outpatient component of the programme.

### 2.3 Outpatient therapeutic care at OTPs

Children (a) who have been discharged from the SC and (b) children with MUAC < 115 mm, with appetite and free of medical complications, are admitted to the OTP, where they receive routine medication, standard therapeutic feeding with RUTF (with a nutrient composition as per WHO standards), and regular visits to the health facility or the *Health Houses* of the *Lady Health Workers* in the community.”

Children attend the OTP site every week or every 2 weeks (depending on the OTP site). At each follow‐up visit, child's weight is monitored and the caregiver of the child is counselled on optimal infant and young child feeding practices, with a primary focus on how to sustain optimal breastfeeding and improve the quality of complementary foods and feeding practices in infants and young children aged 6–23 months. Children are discharged from the OTP when they (a) have completed a minimum of 8 weeks in the programme and (b) have an MUAC ≥ 125 mm for two consecutive visits. Children who are absent for two consecutive visits (if OTP runs every 2 weeks) or three consecutive visits (if OTP runs every week) are defaulters. Children who do not reach the discharge criteria within 16 weeks are discharged as nonrecovered.

To evaluate the effectiveness of Pakistan's CMAM programme, we use a retrospective case series design to analyse programme outcomes in 32,458 children aged 6–59 months old who were admitted to the CMAM programme with an MUAC < 115 mm between January 1 and December 31, 2014. The programme was implemented by the Department of Health, Government of Pakistan, in partnership with eight national and four international NGOs. Data were collected by the implementing partners, both governmental and non‐governmental. Data were reported monthly by all implementing partners using standardized reporting formats. Monthly data reported by implementing partners were collated centrally by the Department of Health, Government of Pakistan, with technical support by UNICEF. The analytical sample (January 1–December 31, 2014) was prepared by an independent data analyst. The Department of Health granted approval to analyse anonymized programme records for the purpose of evaluating programme performance and effectiveness. National and international standards of care are used as benchmarks. Analyses were carried using StataCorp 2011, Stata Statistical Software, Release 12. Mean values are provided as mean ± *SD*; for all tests, *p* < .05 was considered statistically significant.

## 3 RESULTS

At screening, 32,950 children had MUAC < 115 mm: 19,654 (59.6%) were girls, and 28,798 (87.4%) were 6–23 months old (Table 1). Four hundred ninety‐two children (1.5%) with severe congenital/pathological conditions were transferred to the district hospital as their clinical management required highly specialized skills. The remaining 32,458 children (98.5%) were admitted to the CMAM programme (Table 2).

**Table 1:** Characteristics of the children with MUAC < 115 mm at screening

	*N*	%
Girls	19,654	59.6
Boys	13,296	40.4
Total	32,950	100.0
06–11 months old	17,993	54.6
12–23 months old	10,805	32.8
24–35 months old	3,103	9.4
36–59 months old	1,049	3.2
Total	32,950	100.0

*Note*. Pakistan, January 1–December 31, 2014. MUAC = mid‐upper arm circumference.

**Table 2:** Programme outcomes among children admitted to the CMAM programme with MUAC < 115 mm

	*N*	%
MUAC < 115 mm		
Admissions	32,458	98.5
Transfers	492	1.5
Total	32,950	100.0
Admissions		
Deaths	120	0.4
Defaulters	3,456	10.6
Discharged	28,882	89.0
Total	32,458	100.0
Discharged		
Recovered (MUAC ≥ 125 mm)	28,499	98.7
Nonrecovered (MUAC < 125 mm)	383	1.3
Total	28,882	100.0

*Note*. Pakistan, January 1–December 31, 2014. MUAC = mid‐upper arm circumference.

### 3.1 Outcomes among children with MUAC data at admission

The following outcomes were recorded among 32,458 children with MUAC < 115 mm at admission. Deaths: 120 children (0.4%) died while in the programme. Their mean length of stay in the programme was 39.7 ± 24.6 days. Defaulters: 3,456 children (10.6%) defaulted the programme. Their mean length of stay in the programme was 52.8 ± 33.4 days. Discharged: 28,882 children (89.0%) were discharged after a mean length of stay of 69.3 ± 25.7 days and 3.2 ± 3.5 follow‐up visits (Tables 2 and 3).

**Table 3:** Number of treatment visits, mean weight gain, and mean length of stay among children discharged from the CMAM programme

Discharged, all children	
Mean weight gain (g·kg^−1^·day^−1^)	3.2 ± 2.7
Mean number of treatment visits	3.2 ± 3.5
Mean length of stay in programme (days)	69.3 ± 25.7
Discharged, recovered (MUAC ≥ 125 mm)	
Mean weight gain (g·kg^−1^·day^−1^)	3.2 ± 2.6
Mean number of treatment visits	3.2 ± 3.5
Mean length of stay in programme (days)	68.8 ± 25.2
Discharged, nonrecovered (MUAC < 125 mm)	
Mean weight gain (g·kg^−1^·day^−1^)	1.7 ± 2.4
Mean number of treatment visits	4.5 ± 6.1
Mean length of stay in programme (days)	109.3 ± 25.6

*Note*. Pakistan, January 1–December 31, 2014. MUAC = mid‐upper arm circumference.

At discharge, 28,499 children (98.7%) had recovered as their MUAC was ≥125 mm. Their mean length of stay in the programme was 68.8 ± 25.2 days with a mean 3.2 ± 3.5 follow‐up visits. Conversely, 383 children (1.3%) had not recovered (MUAC < 125 mm) despite a significantly longer stay in the programme (109.3 ± 25.6 days) and a significantly higher number of follow‐up visits (4.5 ± 6.1) than children who recovered (MUAC ≥ 125 mm; *p* < .05; Table 3). Children's mean weight gain was 3.2 ± 2.7 g/kg body weight/day. The mean weight gain was greater among children who were discharged recovered (MUAC ≥ 125 mm) than among children who had not recovered (MUAC < 125 mm) at discharge (3.2 ± 2.6 vs. 1.7 ± 2.4 g/kg body weight/day, respectively; *p* < .05).

### 3.2 Outcomes among children with WHZ data at admission

Data on weight and height at admission were available for 11,272 (34.7%) of the children admitted to the programme. One hundred eighty‐one (0.2%) of these children died while in the programme, 1,138 (10.1%) defaulted, and 10,115 (89.7%) were discharged after a mean length of stay of 63.7 ± 20.1 days and a mean 5.2 ± 3.2 follow‐up visits. At discharge, 10,076 children (99.6%) had recovered on the basis of MUAC discharge criteria (MUAC ≥ 125 mm) and 7,720 (82.1%) had recovered on the basis of WHZ criteria (WHZ ≥ −2; Tables 4 and 5).

**Table 4:** Programme outcomes among children admitted to the CMAM programme (MUAC < 115 mm) for whom there was information on WHZ at admission

	All children with WHZ data		Children with WHZ < −3 *SD*		Children with WHZ ≥ −3 *SD*	
	*N*	%	*N*	%	*N*	%
MUAC < 115 mm						
Admissions	11,272	98.4	4,794	98.1	6,478	98.4
Transfers	181	1.6	91	1.9	90	1.6
Total	11,453	100.0	4,885	100.0	6,568	100.0
Admissions						
Deaths	19	0.2	13	0.27	6	0.09
Defaulters	1,138	10.1	465	9.7	673	10.4
Discharged	10,115	89.7	4,316	90.0	5,799	89.5
Total	11,272	100.0	4,794	100.0	6,478	100.0
Discharged						
Recovered (MUAC ≥ 125 mm)	10,076	99.6	4,297	99.6	5,779	99.7
Nonrecovered (MUAC < 125 mm)	39	0.4	19	0.4	20	0.30
Total	10,115	100.0	4,316	100.0	5,799	100.0
Discharged						
Recovered (WHZ ≥ −2)	7,720	76.4	2,118	49.1	5,602	96.7
Nonrecovered (WHZ < −2)	2,387	23.6	2,195	50.9	192	3.3
Total	10,107	100.0	4,313	100.0	5,794	100.0
Implausible cases	8		3		5	

*Note*. Pakistan, January 1–December 31, 2014. MUAC = mid‐upper arm circumference; WHZ = weight‐for‐height *z*‐score.

**Table 5:** Mean weight gain, length of stay, and number of follow‐up visits among children admitted to the CMAM programme (MUAC < 115 mm) for whom there was information WHZ at admission

All children with WHZ data at admission	
Discharged, all children	
Mean weight gain (g·kg^−1^·day^−1^)	3.4 ± 6.8
Mean number of follow‐up visits	5.2 ± 3.2
Mean length of stay in programme (days)	63.7 ± 20.1
Discharged, recovered (MUAC ≥ 125 mm)	
Mean weight gain (g·kg^−1^·day^−1^)	3.4 + 6.9
Mean number of follow‐up visits	5.2 + 3.2
Mean length of stay in programme (days)	63.6 ± 19.9
Discharged, nonrecovered (MUAC < 125 mm)	
Mean weight gain (g·kg^−1^·day^−1^)	1.4 ± 2.3
Mean number of follow‐up visits	7.8 ± 4.7
Mean length of stay in programme (days)	97.4 ± 35.2
Children with WHZ < −3 at admission	
Discharged, all children	
Mean weight gain (g·kg^−1^·day^−1^)	3.5 ± 5.1
Mean number of follow‐up visits	5.1 ± 3.2
Mean length of stay in programme (days)	68.1 ± 22.1
Discharged, recovered (WHZ ≥ −2)	
Mean weight gain (g·kg^−1^·day^−1^)	3.9 ± 6.2
Mean number of follow‐up visits	5.3 ± 3.2
Mean length of stay in programme (days)	67.7 ± 22.3
Discharged, nonrecovered (WHZ < −2)	
Mean weight gain (g·kg^−1^·day^−1^)	2.6 + 1.2
Mean number of follow‐up visits	4.6 ± 3.0
Mean length of stay in programme (days)	68.7 + 22.0
Children with WHZ ≥ −3 at admission	
Discharged, all children	
Mean weight gain (g·kg^−1^·day^−1^)	3.3 ± 7.3
Mean number of follow‐up visits	5.3 ± 3.3
Mean length of stay in programme (days)	60.5 ± 17.6
Discharged, recovered (WHZ ≥ −2)	
Mean weight gain (g·kg^−1^·day^−1^)	3.4 ± 7.4
Mean number of follow‐up visits	5.3 ± 3.3
Mean length of stay in programme (days)	60.6 ± 17.5
Discharged, nonrecovered (WHZ < −2)	
Mean weight gain (g·kg^−1^·day^−1^)	0.7 ± 2.8
Mean number of follow‐up visits	5.4 ± 3.4
Mean length of stay in programme (days)	56.1 ± 20.9

*Note*. Pakistan, January 1–December 31, 2014. MUAC = mid‐upper arm circumference; WHZ = weight‐for‐height *z*‐score.

Among the 11,272 children with data on weight and height at admission, 4,885 (42.7%) had WHZ < −3 whereas 6,568 (57.3%) had WHZ ≥ −3. Death rates were threefold higher among children with WHZ < −3 at admission than among children with WHZ ≥ −3 at admission (0.3% vs. 0.1%; *p* < .05). Discharge rates were similar in both groups (90.0% vs. 89.5%) and so were recovery rates on the basis of MUAC ≥ 125 mm (99.6% vs. 99.7%). Recovery rates on the basis of WHZ > −2 were higher among children with WHZ ≥ −3 at admission than among those with WHZ < −3 at admission (96.7% vs. 49.1%; *p* < .05; Table 4).

Children's mean weight gain during their stay in the programme was 3.4 ± 6.8 g/kg body weight/day. Mean weight gain was higher among children who were discharged recovered (MUAC ≥ 125 mm) than among children who had not recovered (MUAC < 125 mm) at discharge (3.4 ± 6.9 vs. 1.4 ± 2.3 g/kg body weight/day; *p* < .05), whereas the mean length of stay in the programme was longer in children who had not recovered at discharge (MUAC < 125 mm) than among children who had recovered (MUAC ≥ 125 mm) at discharge (97.4 ± 35.2 vs. 63.6 ± 19.9 days; *p* < .05) and so was the mean number of follow‐up visits tended to be greater (7.8 ± 4.7 vs. 5.2 ± 3.2; *p* < .05). Similarly, mean weight gain and length of stay in the programme tended to be higher among children who were admitted with WHZ < −3 and were discharged recovered with WHZ ≥ −2 than among children who were admitted with WHZ < −3 and were discharged nonrecovered with WHZ < −2 (3.9 ± 6.2 vs. 2.6 ± 1.2 g/kg body weight/day; *p* < .05) despite a similar mean length of stay in the programme (67.7 ± 22.3 vs. 68.7 ± 22.0 days; Table 5).

### 3.3 Outcomes among children with HAZ data at admission

Of the 11,272 children for whom data on height and weight were available at admission, 7,825 (69.4%) were stunted (height‐for‐age *z*‐score [HAZ] < −2). Death rates were 2.2‐fold higher among stunted children than among nonstunted children (0.20 vs. 0.09; *p* < .05), whereas default and discharge rates were similar in both groups. Recovery rates on the basis of MUAC (≥125 mm) were similar among stunted and nonstunted children (99.5% vs. 99.8%), whereas recovery rates of the basis of WHZ ≥ −2 were higher among stunted children than among nonstunted children (82.1% vs. 63.4%; *p* < .05; Table 6). Mean weight gain while in the programme tended to be greater among children who were admitted with HAZ < −2 than among children who were admitted with HAZ ≥ −2 both when discharge criteria was MUAC ≥ 125 mm or WHZ ≥ −2 (Table 7).

**Table 6:** Programme outcomes among children admitted to the CMAM programme (MUAC < 115 mm) for whom there was information on HAZ at admission

	All children with HAZ data		Children with HAZ < −2 *SD*		Children with HAZ ≥ −2 *SD*	
	*N*	%	*N*	%	*N*	%
MUAC < 115 mm						
Admissions	11,272	98.4	7,825	98.1	3,447	99.3
Transfers	181	1.6	155	1.9	26	0.7
Total	11,453	100.0	7,980	100.0	3,473	100.0
Admissions						
Deaths	19	0.2	16	0.20	3	0.09
Defaulters	1,138	10.1	797	10.2	341	9.9
Discharged	10,115	89.7	7,012	89.6	3,103	90.0
Total	11,272	100.0	7,825	100.0	3,447	100.0
Discharged						
Recovered (MUAC ≥ 125 mm)	10,076	99.6	6,980	99.5	3,096	99.8
Nonrecovered (MUAC < 125 mm)	39	0.4	32	0.5	7	0.2
Total	10,115	100.0	7,012	100.0	3,103	100.0
Discharged						
Recovered (WHZ ≥ −2)	8,425	83.4	5,753	82.1	1,967	63.4
Nonrecovered (WHZ < −2)	1,682	16.6	1,252	17.9	1,135	36.6
Total	10,107	100.0	7,005	100	3,102	100.0
Implausible cases	8		7		1	

*Note*. Pakistan, January 1–December 31, 2014. MUAC = mid‐upper arm circumference; HAZ = height‐for‐age *z*‐score; WHZ = weight‐for‐height *z*‐score.

**Table 7:** Mean weight gain, length of stay, and number of follow‐up visits among children admitted to the CMAM programme with MUAC < 115 mm and for whom there was information HAZ at admission

Children with HAZ < −2 at admission (stunted children)	
Discharged, all children	
Mean weight gain (g·kg^−1^·day^−1^)	3.5 ± 7.4
Mean number of follow‐up visits	5.3 ± 3.3
Mean length of stay in programme (days)	63.5 ± 20.3
Discharged, recovered (MUAC ≥ 125 mm)	
Mean weight gain (g·kg^−1^·day^−1^)	3.5 ± 7.4
Mean number of follow‐up visits	5.3 ± 3.2
Mean length of stay in programme (days)	63.3 ± 20.0
Discharged, recovered (WHZ ≥ −2)	
Mean weight gain (g·kg^−1^·day^−1^)	3.7 ± 7.4
Mean number of follow‐up visits	5.4 ± 3.3
Mean length of stay in programme (days)	61.8 ± 20.9
Children with HAZ ≥ −2 at admission (nonstunted children)	
Discharged, all children	
Mean weight gain (g·kg^−1^·day^−1^)	3.0 ± 5.3
Mean number of follow‐up visits	4.9 ± 3.2
Mean length of stay in programme (days)	64.2 ± 19.6
Discharged, recovered (MUAC ≥ 125 mm)	
Mean weight gain (g·kg^−1^·day^−1^)	3.0 ± 5.3
Mean number of follow‐up visits	4.9 ± 3.2
Mean length of stay in programme (days)	64.2 ± 19.5
Discharged, recovered (WHZ ≥ −2)	
Mean weight gain (g·kg^−1^·day^−1^)	3.5 ± 6.5
Mean number of follow‐up visits	5.1 ± 3.2
Mean length of stay in programme (days)	62.7 ± 18.0

*Note*. Pakistan, January 1–December 31, 2014. MUAC = mid‐upper arm circumference; HAZ = height‐for‐age *z*‐score; WHZ = weight‐for‐height *z*‐score.

### 3.4 Predictors of death, default, discharge, and recovery (MUAC ≥125 mm or WHZ > −2)

The exposure (independent) variables that were significantly associated with a given outcome (dependent variable) in bivariate analyses were included in linear regression models to identify the main predictors of key outcomes in children admitted to the CMAM programme (Table 8).

**Table 8:** Adjusted odds ratios (AORs) of death, default, discharge, and recovery in children aged 6–59 months by child characteristics

	Death	Defaulter	Discharged	Recovered with MUAC ≥ 125 mm	Recovered with WHZ ≥ −2
All children with MUAC data (*n* = 32,458)					
Age (24–59 vs. 6–23)	2.12*** (1.39–3.24)	1.03 (0.92–1.14)	1.01 (0.91–1.12)	2.06*** (1.37–3.10)	‐
Sex (girls vs. boys)	1.1 (0.76–1.6)	1.08* (1.00–1.60)	0.93** (0.86–1.00)	0.77*** (0.62–0.95)	‐
All children with WHZ data (*n* = 11,272)					
Age (6–23 vs. 24–59)	0.72 (0.46–4.26)	0.93 (0.78–1.10)	1.09 (0.91–1.30)	0.48 (0.15–1.56)	2.17*** (1.89–2.51)
Sex (girls vs. boys)	1.39 (0.54–3.59)	0.97 (0.85–1.10)	1.02 (0.90–1.16)	0.72 (0.36–1.41)	1.28*** (1.16–1.42)
WHZ admission (<−3 vs. ≥−3)	3.43*** (1.28–9.21)	0.92 (0.81–1.05)	1.06 (0.93–1.20)	0.65 (0.34–1.24)	0.06*** (0.05–0.07)
HAZ admission (<−2 vs. ≥−2)	2.86* (0.82–10.02)	1.01 (0.88–1.16)	0.97 (0.85–1.12)	0.42** (0.18–0.97)	2.00*** (1.79–2.22)

*Note*. Pakistan, January 1–December 31, 2014. AOR with standard errors in parentheses.

* *p* < .1.

** *p* < .05.

*** *p* < .01.

Multivariate regression analysis indicates that the odds of death were higher among children aged 24–59 months (adjusted odds ratio [OR] = 2.12; 95% CI [1.39, 3.24]; *p* < .001), children with WHZ < −3 at admission (adjusted OR = 3.43; 95% CI [1.28, 9.21]; *p* < .001), and children with HAZ < −2 at admission (adjusted OR = 2.86; 95% CI [0.82, 10.02]; *p* < .1). Default rates were higher among girls than among boys (adjusted OR = 1.08; 95% CI [1.00, 1.60]; *p* < .1), whereas discharge rates were significantly lower among girls than among boys (adjusted OR = 0.93; 95% CI [0.86, 1.00]; *p* < .05). Recovery rates on the basis of MUAC ≥ 125 mm at discharge were significantly higher among children aged 24–59 months (adjusted OR = 2.06; 95% CI [1.37, 3.10]; *p* < .001), boys (adjusted OR = 1.30; 95% CI [1.05, 1.61]; *p* < .001), and children admitted with HAZ ≥ −2 (adjusted OR = 2.37; 95% CI [1.03, 5.44]; *p* < .05). Recovery rates on the basis of WHZ ≥ −2 at discharge were significantly higher among children aged 6–23 months (adjusted OR = 2.17; 95% CI [1.89, 2.50]; *p* < .001), girls (adjusted OR = 1.28; 95% CI [1.16, 1.42]; *p* < .001), children admitted with WHZ ≥ −3 (adjusted OR = 17.2; 95% CI [15.3, 19.3]; *p* < .001), and children admitted with HAZ < −2 (adjusted OR = 2.00; 95% CI [1.8, 2.2]; *p* < .001; Table 8).

## 4 DISCUSSION

In this paper, we analyse programme outcomes in 32,458 children aged 6–59 months discharged from Pakistan's CMAM programme between January 1 and December 31, 2014. At admission, all children had a MUAC < 115 mm. Two salient features of children admitted to the CMAM programme are their gender (60% girls) and young age (87.4% in the age group 6–23 months). Strikingly similar figures have been reported in India (Aguayo et al., 2013; Aguayo et al., 2014; Burza et al., 2015). Therefore, CMAM programmes in Pakistan need to give priority to the early detection of SAM in children under 2 years of age and may need to pay particular attention to the nutrition situation of girls.

The programme achieved survival outcomes (0.4% child deaths) that compare favourably with national (<3% child deaths) and international (<10% child deaths) standards of care (Government of Pakistan, 2014; Sphere Project, 2011). This is an important finding as the primary objective of the CMAM programme is to reduce fatality rates among children with SAM. Children with severe congenital or pathological conditions (*n* = 492; 1.5%) were transferred to the district hospital and were not transferred back to the CMAM programme. In a “worst case scenario” analysis (i.e., these 492 children stayed in the CMAM programme and died), the death rate in the programme (1.9%) would still be below the national standard of care. Mortality rates observed in Pakistan's CMAM programme are comparable with those reported in CMAM programmes in India (Aguayo et al., 2013; Burza et al., 2015) and lower than those observed in CMAM programmes elsewhere (Collins et al., 2006). Reasons for this apparent lower mortality among children with SAM in Pakistan and India deserves further investigation.

The proportion of defaulters (10.6%) is lower than national and international standards of care (<15%) and lower than the default rates reported by programmes in India, where the proportion of defaulters ranged from 32% to 38% (Aguayo et al., 2013; Burza et al., 2015). Outcomes among children who defaulted the programme are unknown. Recovery rates on the basis of MUAC ≥ 125 mm (87.8% of the admitted and 98.7% of the discharged) were above national and international standards of care (>75%). Mean weight gain while in the programme (3.2 ± 2.7 g/kg body weight/day), which was slightly lower in CMAM programmes elsewhere (4–5 g/kg body weight/day; Burza et al., 2015; Collins et al., 2006), combined with a mean length of stay of about 10 weeks (7.3 ± 5.6 and 8.7 ± 6.1 weeks in India; Burza et al., 2015) resulted in recovery rates that were higher than those reported by CMAM programmes in India, where the proportion of children discharged recovered ranged from 53.4% to 65.0% (Aguayo et al., 2013; Burza et al., 2015).

Our findings confirm that appropriate RUTF—with a nutrient composition as per WHO recommendations—are well accepted by South Asian children (Dube et al., 2009), are effective in supporting catch‐up growth in children with SAM (Diop, Dossou, Ndour, Briend, & Wade, 2003), can be safely used in CMAM programmes (Collins et al., 2006), can ensure survival rates >95%, and can bring about recovery rates of up to 90% (Diop et al., 2003).

In most populations, children identified as severely wasted by MUAC will generally be at higher risk of death than those identified by WHZ (Briend, Maire, Fontaine, & Garenne, 2012). It has been recommended that where resources are limited, it is preferable to screen by MUAC than by WHZ (Myatt, Khara, & Collins, 2006). However, global evidence also indicates that when children with SAM are identified on the basis of both MUAC < 115 mm or WHZ < −3, the populations identified by these two criteria do not overlap uniformly (Trehan & Manary, 2015). Further, some researchers have alerted that a significant number of children in need could be missed when MUAC is used as the only admission criteria and recommend that both MUAC and WHZ be used (Grellety & Golden, 2016).

Pakistan's CMAM programme uses MUAC as the primary criteria for the diagnosis and admission of children with SAM. Children with MUAC < 115 mm are admitted to the programme and are discharged when MUAC ≥ 125 mm as recommended by WHO (2013a). In our analytical sample, only 42.5% of the children with MUAC < 115 mm had WHZ < −3. Children with both MUAC < 115 mm and WHZ < −3 at admission had poorer survival outcomes (i.e., threefold higher death rates) than children with MUAC < 115 mm but WHZ ≥ −3 at admission. Furthermore, only 49.1% of children with WHZ < −3 at admission had WHZ ≥ −2 at discharge (and therefore were not wasted anymore) compared with 96.7% among children with WHZ ≥ −3 at admission.

We looked at differential programme outcomes among stunted and nonstunted children. About 70% of the children in our analytical sample were stunted (HAZ < −2). Children who were stunted at admission had poorer survival outcomes (i.e., 2.2‐fold higher death rates) than children who were not stunted (HAZ ≥ −2) when they were admitted, which is in line with previous research findings indicating higher mortality rates among stunted children. However, recovery rates on the basis of WHZ ≥ −2 at discharge were higher among stunted children than among nonstunted children (82.1% vs. 63.4%, respectively). Reasons for these differential recovery rates between stunted and nonstunted rates deserve future attention.

Multivariate regression analysis indicates that the odds of death were higher among children with WHZ < −3 and/or HAZ < −2 at admission indicating that children with indicators of anthropometric failure other than MUAC < 125 mm (i.e., WHZ < −3 and/or HAZ < −2) were at a significantly higher risk of death. The odds of recovery on the basis of MUAC ≥ 125 mm were higher among children with HAZ ≥ −2 at admission, whereas the odds of recovery on the basis of WHZ ≥ −2 were significantly higher among children with WHZ ≥ −3 and/or HAZ < −2 at admission.

South Asia is the epicentre of the global burden of wasting. However, the coverage of effective interventions remains unacceptably low (UNICEF, 2013). There is growing consensus among scientists and practitioners that South Asian countries need to recognize SAM as a major public health problem and mobilize internal resources for its management (Ahmed et al., 2014). Particular attention needs to be given to the detection of children with SAM as early as possible, when children are younger and less severely wasted (WHO & UNICEF, 2009). In areas where active surveillance and case finding are conducted effectively, the vast majority of children with SAM are identified early in their illness, have no medical complications, and can be treated in their communities as outpatients (Trehan & Manary, 2015). Over 50 countries have adopted CMAM to address SAM as a public health problem (Field Exchange, 2012).

The use of appropriate RUTF (i.e., in line with the composition recommended by WHO) is key to ensure adequate weight gain and full recovery (Golden & Briend, 1993). RUTF should preferably be produced from locally available ingredients. Appropriate RUTF for CMAM are now manufactured to international quality standards in a good number of low‐ and middle‐income countries, including India. Countries that do not have the capacity to produce RUTF from locally available food ingredients can benefit from other countries in South Asia that can produce it. Consensus is strong among South Asian researchers and practitioners that CMAM for children with uncomplicated SAM is a promising alternative (Ahmed et al., 2014; Kapil, 2009; Kapil & Sachdev, 2010; Prasad, Sinha, & Sridhar, 2012; Sachdev, Kapil, & Vir, 2010; Swaminathan, 2009).

## 5 CONCLUSION

Our analysis shows that Pakistan's CMAM programme is effective in achieving good survival and recovery rates. Population‐level impact can be increased by placing greater emphasis on children 6–23 months old, children with multiple anthropometric failure, and girls and by scaling up CMAM in the provinces and areas where the risk, prevalence, and/or burden of SAM is highest. The experience in Pakistan demonstrates that governments and their partners can strengthen the existing health systems with situation‐specific adjustments to provide timely and quality care for children with SAM through an integrated approach that combines facility‐based therapeutic care and CMAM.

## CONFLICTS OF INTEREST

The authors declare that they have no conflicts of interest. The opinions expressed on this paper are those of the authors and do not necessarily represent an official position of the organizations that they are affiliated with.

## CONTRIBUTIONS

VMA designed the study, led data analysis, and wrote the paper; NB led data management; SSQ, ANG, MMA, NN, and MG contributed to data interpretation and paper writing. All authors have read and approved the final manuscript.

## SOURCE OF FUNDING

United Nations Children's Fund (UNICEF) Regional Office for South Asia (ROSA).
